# Importance of Adapting the Surgical Technique to the Characteristics of the Breast Implant Surface Type

**DOI:** 10.1093/asj/sjae189

**Published:** 2024-09-18

**Authors:** Nenad Stepic

I would like to comment on the study titled Comparison of Polytech MESMO and Motiva Ergonomix Breast Implants with a Focus on Displacement Issues: A Single Surgeon's Experience in 329 Patients, which compares complication rates of 2 different styles of implants: a round, 4-micron surface implant, and an anatomical textured implant.^1^ There are points made by the author in this article that deserve additional discussion, including study design and conclusions that Motiva implants have a higher rate of complications.

Of primary concern is the author's lack of acknowledgment that MESMO has a higher number of implant removals than Motiva (11 vs 10). Given that all removal procedures involve reoperation, the reoperations reported in Table 2 should be corrected from 1 and 6 to 12 and 16 (for MESMO and Motiva, respectively). After updating the analysis, the resulting *P* value is no longer statistically significant; therefore, the reoperation conclusions are not accurate. In addition, the conclusion that Motiva has higher rates of “overall displacement issues” is based on a composite category of 3 events (bottoming out, high riding, and rotation); however the author does not similarly discuss MESMO's displacement issues (rotation and high riding, of which Motiva had zero).

The author states regarding the incidence of complications that “this paper could not capture . . . [such as] capsular contracture, implant rupture, and BIA-ALCL.” It is a limitation not to include these outcome rates, because most surgeon and patient concerns are focused on safety issues, of which these 3 events are paramount. With 329 patients in their study, information on those 3 complications would have added meaningful data to this article for surgeons to utilize when assessing the implants. Similarly, related to safety, the author did not discuss the manufacturing process of MESMO implants, which are manufactured with the use of salt, termed “salt-loss.” This method is similar to that employed for Allergan's Biocell, which was removed from the market due to clinical inflammatory complications associated with this type of surface manufacturing, including biofilm formation, silicone debris shedding, friction tissue damage, double-capsule, capsular contracture, and, importantly, breast implant–associated anaplastic large cell lymphoma (BIA-ALCL).^2-4^

I would encourage the author to publish an update to the study time frame so that it renders accurate complication rates of MESMO implants. As currently written, the complication rates appear premature due to the lack of longer-term, evidence-based clinical outcomes. This paper reported the median follow-up as 8 months. However, statistical analyses were performed at 12 and 24 months, and figures provided data out to 60 months, leading to confusion about the length of follow-up and the time to outcomes. In the discussion, the author acknowledges that some of the “bottoming out” complications are “the result of improper surgical maneuver[ing],” yet does not clarify the temporal distribution of the implantations (how many Motiva vs MEMSO were implanted in each of the 6 enrollment years).

There also appear to be surgical inconsistencies. The author describes performing the same technique for both Motiva and MESMO implants, while acknowledging in the discussion that “a different technique must be used for smooth implants than [for] textured.” It is inherently understood that if a surgeon does not adjust technique from that for a textured to that for a smoother implant, pocket position issues will, in turn, increase. Further, the author's protocol fixes the inframammary fold, a technique that is more commonly utilized for textured anatomical implants, not for smooth implants. Inevitably, when utilizing a textured implant technique to perform smooth implant surgeries, the author sets himself up for later challenges.

Finally, there are a limited number of published clinical studies about the MESMO implant, and the 3 that are published are authored by Polytech employees and a paid consultant, including this paper.^5,6^ One of the other 3 studies is a patient survey with low (6.6%) patient follow-up, therefore outcome data are not valid.^5^ Conversely, the Motiva devices have many peer-reviewed, independent studies, publicly posted post–market surveillance data from more than 13 years, and recently published 4-year FDA clinical study data, all of which present the same low device-related complication rates and under 1% rates of capsular contracture and rupture.^7,8^ In my own experience (with no conflicts of interest) with both of these devices over the past 12 years and long-term data representing 1229 Polytech devices and 1038 Motiva devices, the displacement with Polytech implants is evidently higher than with Motiva implants, with rates of 5.8% (range 4.4%-7.1%) and 0.8% (range 0.2%-1.2%), respectively.

I believe that the data presentation and conclusions in the article described above will cause confusion for readers. The paper may have been more informative if the author had compared a textured device with another textured device. Unfortunately, as currently written, the paper will create confusion to the growing number of surgeons who avoid textured devices and have few complications and high patient satisfaction.

## References

[1] Montemurro P . Comparison of polytech MESMO and Motiva Ergonomix breast implants with a focus on displacement issues: a single surgeon’s experience with 329 patients. Aesthet Surg J. 2024;44(9):948–956. doi: 10.1093/asj/sjae071 38547508

[2] Mempin M, Hu H, Chowdhury D, Deva A, Vickery K. The A, B and C’s of silicone breast implants: anaplastic large cell lymphoma, biofilm and capsular contracture. Materials. 2018;11(12):2393. doi: 10.3390/ma11122393 30486500 PMC6316940

[3] Marinkovic M, Nikolic J, Gusman V, Jovanovic M, Rasovic P. Silicon breast implants’ texture affecting bacterial biofilm formation. Srp Arh Celok Lek. 2022;150(7-8):439–444. doi: 10.2298/SARH211012065M

[4] U.S. Food and Drug Administration . Company announcement. Allergan voluntarily recalls BIOCELL^®^ textured breast implants and tissue expanders. July 24th, 2019. Accessed May 28th, 2024. https://www.fda.gov/safety/recalls-market-withdrawals-safety-alerts/allergan-voluntarily-recalls-biocellr-textured-breast-implants-and-tissue-expanders

[5] Montemurro P, Siri G, Clerico L. “Safety and performance of POLYTECH MESMO breast implants: a 5-year post-market surveillance study on 919 patients.” Aesthet Surg J Open Forum. 2022;4:ojac011. doi: 10.1093/asjof/ojac011 35415603 PMC8994747

[6] Montemurro P, Pietruski P. Twelve years and over 2400 implants later: augmentation mammoplasty risk factors based on a single plastic surgeon’s experience. Plast Reconstr Surg Glob Open. 2024;12(4):e5720. doi: 10.1097/GOX.0000000000005720 38596589 PMC11000759

[7] Aitzetmuller-Kleitz ML, Yang S, Wiebinghaus P, et al Complication rates after breast surgery with the Motiva smooth silk surface silicone gel implants—a systematic review and meta-analysis. J Clin Med. 2023;12:1881. doi: 10.3390/jcm12051881 36902667 PMC10004118

[8] Establishment Labs Holdings Corp . Establishment Labs notes presentation of 4-year results from Motiva U.S. IDE study at the aesthetic meeting 2024. Published May 2nd, 2024. Accessed May 28th, 2024. https://investors.establishmentlabs.com/press-releases/press-releases-details/2024/Establishment-Labs-Notes-Presentation-of-4-Year-Results-from-Motiva-U.S.-IDE-Study-at-The-Aesthetic-Meeting-2024/default.aspx

